# Atypical SARS in Geriatric Patient

**DOI:** 10.3201/eid1002.030322

**Published:** 2004-02

**Authors:** Augustine K.H. Tee, Helen M.L. Oh, K.P. Hui, Christopher T.C. Lien, K. Narendran, B.H. Heng, A.E. Ling

**Affiliations:** * Changi General Hospital, Singapore; † National Healthcare Group, Singapore; ‡ Singapore General Hospital, Pathology, Singapore

**Keywords:** severe acute respiratory syndrome

## Abstract

We describe an atypical presentation of severe acute respiratory syndrome (SARS) in a geriatric patient with multiple coexisting conditions. Interpretation of radiographic changes was confounded by cardiac failure, with resolution of fever causing delayed diagnosis and a cluster of cases. SARS should be considered even if a contact history is unavailable, during an ongoing outbreak.

The recent discovery of the novel severe acute respiratory syndrome–associated coronavirus (SARS-CoV) responsible for the outbreak of SARS (*1*–*3*) in China, Hong Kong, Vietnam, Singapore, Canada, and Taiwan has caused concern among the medical community because it spreads easily within the hospital environment. An unprecedented cooperative effort by the international medical research community has seen the rapid development of laboratory tests consisting of polymerase chain reaction (PCR), antibody testing, and virus isolation (*4*). However, before these tests were widely available, the disease was diagnosed on the basis of its clinical presentation, according to the World Health Organization (WHO) case definition (*5*). The presence of a fever of more than 38°C, essential and sentinel in the detection of SARS, has been described in papers from Hong Kong and Canada (*1*,*6*–*8*).

Nevertheless, these surveillance case definitions may not be sufficiently sensitive (*9*) as clinical features and epidemiologic case definitions may not coincide perfectly (*10*). We describe a case of SARS (with delayed diagnosis) and a consequent cluster of cases that resulted because of difficulty in establishing a positive contact history and atypical signs and symptoms.

## Case Report

The patient was a 90-year-old Singaporean Chinese woman who was a resident of a nursing home. She had a past history of vascular dementia with dysphagia and behavioral abnormalities, ischemic heart disease with atrial fibrillation, and congestive cardiac failure. In addition, she also suffered from type 2 diabetes mellitus, hypertension, osteoporosis, bilateral osteoarthritis of the knees, and an old traumatic fracture of the left humeral neck. As such, she was fully dependent in her daily activities.

**Figure 1:**
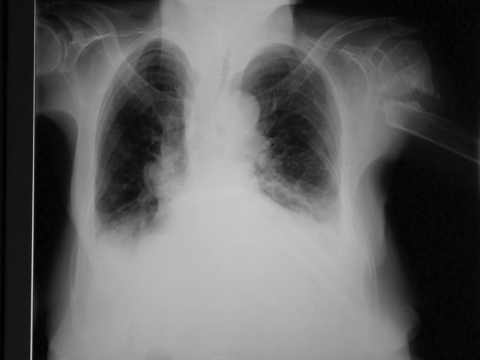
Chest radiograph at first admission.

She was admitted to the geriatric department of Tan Tock Seng Hospital (*11*) on March 7, 2003, for pneumonia and urinary tract infection. These infections responded to a course of intravenous antimicrobial drugs. She also was assessed to have mild dysphagia, which required thickened fluids and blended diet without nasogastric feeding. Her chest radiograph before discharge showed persistent bilateral lower zone consolidation (Figure 1), consistent with bilateral crepitations on auscultation. However, the patient was afebrile and improved functionally to being ambulant with assistance. She was discharged to the nursing home on March 20.

Within the next two days, the patient progressively became breathless, with nausea and vomiting. There was no associated cough or diarrhea. She was eventually admitted to the medical department of Changi General Hospital, a designated non-SARS hospital, on March 25. On admission to the isolation room, she had a maximal tympanic temperature of 38.3°C, with defervescence the next day. She remained afebrile during the remainder of her stay. Her blood pressure was 124/84 mm Hg, pulse rate of 96 beats per minute, and respiratory rate of 32 breaths per minute. Her pulse oximetry was 100% while on 4 L per minute of intranasal oxygen. The jugular venous pressure was not elevated. Bilateral basal crepitations were heard on examination. All healthcare workers attending to the patient wore the recommended personal protective equipment, including gown, gloves, and N95 respirators, each time they entered the isolation room.

Investigations on admission showed that the patient’s hemoglobin was 10.6 g/dL, leukocyte count was 7,200/mm^3^ (86.3% polymorphs, 8.6% lymphocytes), and platelet count was 304,000/mm^3^. The serum urea was 6.6 mmol/L; serum potassium, 5.0 mmol/L; serum sodium, 138 mmol/L; and creatinine, 79 μmol/L. The liver function tests showed a total bilirubin, 10.6 μmol/L; serum albumin, 30 g/L, serum alkaline phosphatase, 106 μ/L; serum alanine transaminase, 16 μ/L; serum aspartate transaminase, 33 μ/L. Her creatine kinase was 45 μ/L, and C-reactive protein was elevated at 147.0 mg/L. She was diagnosed to have aspiration pneumonia, and intravenous ceftriaxone and metronidazole were prescribed. Her chest radiograph showed infiltrates in the right lower zone. Her urine, sputum, and blood cultures did not yield any bacterial growth. Serologic testing for *Mycoplasma*, *Legionella*, and *Chlamydia* and nasopharyngeal aspirate for common viral antigens were not performed, as clinical suspicion was low. She was subsequently transferred to the geriatric unit. Her condition improved, and she was placed in the general ward on March 28. No protective equipment was used by staff attending her in the general ward. It was ascertained that she was previously admitted to a non-SARS ward in Tan Tock Seng Hospital.

**Figure 2:**
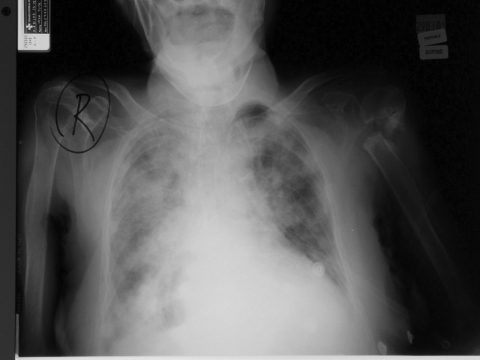
Repeat chest radiograph as second admission.

However, on March 29, the patient became restless and more breathless. A repeat chest radiograph (Figure 2) confirmed congestive cardiac failure. Her repeat leukocyte count was 8,800/mm^3^ (93.0% polymorphs, 4.5% lymphocytes), and the platelet count was 167,000/mm^3^. There was mild hyponatremia (133 mmol/L) and worsening C-reactive protein levels (179.9 mg/L) but a stable creatine kinase (50 u/L).

### Intravenous diuretic therapy was instituted, but in view of her poor premorbid functional status, the patient was not intubated or moved to in an intensive care unit. She went into respiratory failure and died on March 30. Death was certified as being caused by pneumonia, with a contributing factor of ischemic heart disease. No autopsy or postmortem specimens were taken.

### In the week after the patient’s death, a cluster of cases of atypical pneumonia surfaced, all of which could be traced to this patient. Pneumonia developed in the patient’s daughter-in-law, who had visited her in the hospital, and two grandsons living in the same household as the daughter-in-law. Another son-in-law, who met this daughter-in-law during the funeral, also contracted a respiratory illness. A healthcare worker, who was unprotected while caring for the patient, was also admitted to Changi General Hospital for severe pneumonia. He was later transferred to Tan Tock Seng Hospital where he was diagnosed with SARS. He required prolonged mechanical ventilation and eventually died of the illness. A female hospital cleaner in Changi General Hospital, who cleaned the room and tidied the patient’s bed in the general ward, became symptomatic 3 days after the patient died. She was admitted to Changi General Hospital 10 days later and was transferred to Tan Tock Seng Hospital the next day. Her husband was subsequently admitted to Tan Tock Seng Hospital with SARS. All cases in the cluster had fever as a presenting complaint. On the basis of epidemiologic data (contact tracing linking her to one of the three original index cases in Singapore) (*12*), the index patient’s cause of death was determined to be SARS (Figure 3). Serologic testing for SARS-CoV by using enzyme-linked immunosorbent assay (ELISA) techniques on various specimens during admission for febrile illness were positive at titers of 400 to 6,400 for all patients within the cluster except the patient’s daughter-in-law and the healthcare worker from the nursing home.

**Figure 3:**
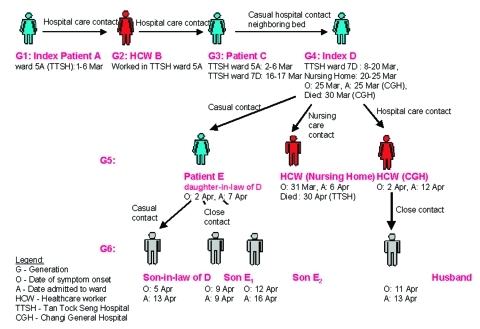
Cases linked to index D.

## Conclusions

Since the issue of a global alert on atypical pneumonia by the World Health Organization on March 12, reported cases of SARS increased daily and appeared in other countries, including Canada, the United States, Europe, and Africa. The first three cases in Singapore were reported on March 13. These cases were traced to a doctor from Guangdong who infected 13 guests at a Hong Kong hotel (*13*). The clinical features of SARS are fairly nonspecific with a body temperature of >38°C, occurring in 100% of patients, being the most sensitive feature in all the case series published thus far (*6*–*8*). Other symptoms described thus far have included nonproductive cough, dyspnea, malaise, diarrhea, chest pain, headache, myalgia, and vomiting.

We describe here a fairly complicated atypical signs and symptoms of SARS in an elderly patient. The patient had a fever, which responded to a course of broad-spectrum antimicrobial drugs, thus behaving in a manner not much different from a typical community-acquired pneumonia. The absence of fever during the final course of the patient’s hospitalization could have been caused by an altered immune response in the geriatric age group, with a resulting normal leukocyte count. Furthermore, prior usage of antimicrobial drugs and possible aspiration from dysphagia may further complicate detection of the disease. The suspicion of SARS in this case was thus low before eventual epidemiologic links were established retrospectively. Dyspnea is a common symptom reported previously, ranging from 60% to 80% of patients. Cough has also been noted in 80% to 100% of cases in previous studies (*6*,*8*). However the absence of cough, especially in the elderly, could be due to an underlying weak cough reflex. Vomiting, though present in our patient, was only accounted for in 10% of cases in the Canadian series (*8*). In a frail older person, this could also be caused by a number of circumstances.

Our patient had characteristic lymphopenia, which was seen in about 90% of reported cases. In addition, she also had mild hyponatremia and elevated C-reactive protein. However, thrombocytopenia, elevated transaminases, or raised creatine kinase levels were absent.

Serial chest radiograph progressed from a predominantly right lower lobe patchy consolidation to a radiographic picture of congestive cardiac failure. Reports from SARS cases have described mainly basal lung opacities, without any pleural effusion. An underlying poor cardiac function may masquerade the true picture of the air space disease characteristic of SARS, especially if the stress of infection decompensates left ventricular ejection fraction. This radiologic interpretation could potentially mislead clinicians and lead to more patients, family members, and healthcare workers becoming infected. In addition, a bimodal pattern of time to deterioration of clinical symptoms has been previously reported (*14*).

The information currently available on transmission of SARS has been attributed to respiratory droplets from close contact which has been defined by WHO to be having cared for, having lived with, or having direct contact with respiratory secretions or body fluids of a patient known to be a suspected SARS case. As the patient lived in a nursing home, the brief social contact during visits by family and friends, may prove sufficient for transmitting the virus.

Furthermore, the issue of possible coinfection and the influence of coexisting conditions have not been thoroughly investigated, which may change the clinical picture of SARS so as to conceal detection. Uncharacteristic clinical signs and symptoms, without any travel or contact history, are difficult to recognize.

Our case serves to highlight atypical signs and symptoms of SARS, especially the resolving fever, delay in establishing a positive contact history, and the nonspecific chest radiographic appearance that could be affected by concurrent coexisting conditions, such as cardiac failure. We wish to draw attention to clinicians, so that a high level of suspicion is present as the SARS-CoV is highly contagious and can cause severe disease. We observed that despite being cared for in the general ward by staff without full personal protective equipment, only one healthcare worker in Changi General Hospital was infected. This observation supports the hypothesis that the virus may not transmit effectively under certain conditions. Nevertheless, late diagnosis may lead to large clusters, as delayed isolation of suspect cases increases the risk of onward transmission in the community (*15*). A positive contact history may not be obvious, particularly in patients with cognitive impairment, until retrospective analysis is done. There is thus a need for continued surveillance of fever and clusters of pneumonia cases to improve the chances of early detection. Nonetheless, with the imminent availability of accurate and rapid diagnostic tests, there is hope that the diagnosis of SARS can be made with more certainty. This could be further enhanced by a revised case definition.
